# Liquid biopsy and non-small cell lung cancer: are we looking at the tip of the iceberg?

**DOI:** 10.1038/s41416-022-01777-8

**Published:** 2022-03-09

**Authors:** Laura Bonanno, Alessandro Dal Maso, Alberto Pavan, Elisabetta Zulato, Lorenzo Calvetti, Giulia Pasello, Valentina Guarneri, PierFranco Conte, Stefano Indraccolo

**Affiliations:** 1 Medical Oncology 2, Istituto Oncologico Veneto IOV—IRCCS, Padova, Italy; 2 Department of Surgery, Oncology and Gastroenterology, University of Padova, Padova, Italy; 3 Medical Oncology Department, AULSS 3 Serenissima, Mestre-Venezia, Italy; 4 Basic and Translational Oncology, Istituto Oncologico Veneto IOV—IRCCS, Padova, Italy; 5 Medical Oncology Department, San Bortolo General Hospital, AULSS 8 Berica East District, Vicenza, Italy

**Keywords:** Non-small-cell lung cancer, Tumour biomarkers

## Abstract

The possibility to analyse the tumour genetic material shed in the blood is undoubtedly one of the main achievements of translational research in the latest years. In the modern clinical management of advanced non-small cell lung cancer, molecular characterisation plays an essential role. In parallel, immunotherapy is widely employed, but reliable predictive markers are not available yet. Liquid biopsy has the potential to face the two issues and to increase its role in advanced NSCLC in the next future. The aim of this review is to summarise the main clinical applications of liquid biopsy in advanced non-small cell lung cancer, underlining both its potential and limitations from a clinically driven perspective.

## Introduction

The role of precision medicine in advanced non-small cell lung cancer (NSCLC) has been continuously increasing in the latest years. The path to achieve complete molecular information is certainly long and challenging, as it is affected by the evolving and ever-changing nature of the neoplastic phenomenon itself. Cells mutability, metabolic changes and micro-environmental features defining different metastatic sites are able to influence the spawn and the growth of divergent tumoural sub-clones [1, 2], while cancer therapy itself affects clonal selection and host-tumour interaction [3].

Ideally, molecular information should derive from different anatomic sites, both at baseline and recurrence. In daily practice, however, tumour accessibility and patients’ clinical conditions often dictate the timing, quantity and final quality of tumour sampling [4, 5]. Molecular characterisation obtained from tissue biopsy is intrinsically partial and often not feasible due to low quantity or poor quality tumour DNA.

Tumour cells are able to shed macromolecules into the bloodstream, both from primary and metastatic sites. Assays capable of sampling, isolating and testing analytes from a biological fluid are referred to as a liquid biopsy. Liquid biopsy is minimally invasive and easily repeatable. Various biologic analytes could be isolated from peripheral blood, i.e. circulating tumour DNA (ctDNA), circulating tumour cells (CTCs), circulating exosomes, platelet RNA and ctRNA. ctDNA is certainly the most studied one [6, 7]. Cell-free DNA (cfDNA) in blood refers to degraded DNA fragments, derived mostly from normal white blood cells and stromal cells; in cancer patients, a fraction of cfDNA is represented by ctDNA, released from tumour cells through apoptotic and necrotic cell death or via active processes [8, 9]. ctDNA is amenable to be tested for the presence of somatic mutations, gene copy number variations (CNVs) and gene rearrangements and has a good concordance rate with tissue analysis [10–13].

**Fig. 1:**
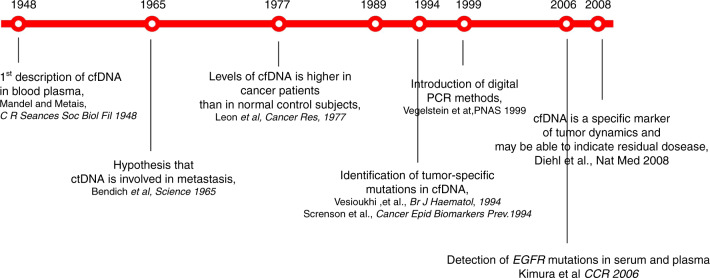
Timeline of the development of liquid biopsy. Development of liquid biopsy, from the discovery of cell-free DNA in plasma (cfDNA) to the capacity of detecting and analyzing tumour-associated genetic alterations.

Historically, the first clinical application of liquid biopsy in advanced NSCLC was the detection of sensitising *EGFR* mutations [14–19] (Fig. 1). These pioneer studies were followed by the advent and widespread application of high-throughput sequencing methods, such as next-generation sequencing (NGS), which broadened our capacity for genomic profiling. NGS widened the spectrum of detection of somatic mutations from ctDNA, leading to different opportunities. First, whenever a druggable alteration was found, matched therapy might be offered [20–22]. Second, the presence of certain mutations or co-mutations could provide prognostic and predictive information [23, 24]. Last, the detection of tumour-specific genetic alterations at baseline and the measurement of their variation during treatment could be useful to monitor the course of the disease [25–27]. Large (>1.6 Mb) NGS assays allow also quantification of the tumour mutational burden (TMB), which is the total number of somatic mutations per coding area of a tumour genome. TMB is considered as a surrogate for tumour neo-antigen load and therefore a potential predictive marker for immune checkpoint inhibitors (ICIs) [28–31].

Considering the feasibility of liquid biopsy as a diagnostic tool and the huge amount of data obtainable, it is crucial to integrate this technique into daily clinical workflow.

The aim of this review is to analyse currently available data and the main ongoing clinical trials, in order to provide a view on potential future applications in the clinical practice of advanced NSCLC.

## The achievements of yesterday: from the discovery of cfDNA to the ability to characterise tumours in plasma

The main historical steps at the basis of the development of liquid biopsy as a tool in clinical practice are summarised in Fig. 1.

The presence of nucleic acids in the circulation was first reported by Mandel and Metais in 1948 [32]. Since then, many studies have reported a relative high concentrations of cell-free nucleic acid in the blood of cancer patients [33, 34]. Different mechanisms have been proposed concerning the source of ctDNA: cell lysis, breakdown of circulating tumour cells, destruction of tumour micro-metastases and active release by tumour cells [35]. ctDNA is frequently highly diluted in plasma and its total concentration is also influenced by the clearance and degradation by the nuclease activity.

After the discovery of ctDNA and its likely correlation with metastatic potential, research efforts mainly focused on the characterisation of tumour-associated genetic alterations in plasma. In this context, highly sensitive analytical techniques, as well as stringent and standardised pre-analytical procedures, are needed, in order to face the short half-life of cfDNA and the detection of low-frequency somatic mutations against a high background level of wild-type cfDNA fragments. The recommended workflow for plasma management considers the timing for its collection particularly crucial, as the contamination with DNA from leukocytes occurs if blood remains unprocessed for a long time (up to 4 h) [36, 37]. In order to overcome the need for rapid processing of plasma samples, commercial cell stabiliser tubes have been recently developed to prevent white blood cells degradation and to inhibit nuclease activity up to several days after blood draw [38]. Moreover, in order to safeguard the cfDNA stability and prevent its degradation in ex vivo plasma, immediate cooling at 4 °C and then storage in frozen conditions are strictly required to minimise nuclease activity [36].

Over time, various analytical methods have been developed and applied for the molecular characterisation of ctDNA. They can be divided into narrow and broad approaches.

The former methods involve assays that are able to detect genetic alterations in selected regions of cfDNA and are PCR-based techniques. A prominent PCR-based platform is the cobas *EGFR* mutation test (Roche diagnostics): a real-time PCR (rt-PCR) test able to provide quantitative information about the presence of specific *EGFR* gene alterations [39–41]. This assay is currently approved by U.S. Food and Drug Administration (FDA) for the detection of common sensitising *EGFR* alterations and of acquired resistance mutations T790M in ctDNA. Although not FDA approved, highly sensitive assays, such as the droplet digital PCR (ddPCR), can also detect additional genetic alterations in ctDNA with higher sensitivity and specificity [26, 42].

ddPCR is an approach based on water-oil emulsion droplet technology: sample is partitioned so that each droplet has from one to five molecules of DNA, and PCR amplification of the template molecules occurs in each individual droplet [43]. Such methods have several pros; the most important ones are sensitivity, affordability and short turn-around time. Notably, their analytical specificity reaches 98% [39–41]. Anyway, even though the detection of *EGFR* mutations in ctDNA is possible even at low mutated allele abundance (generally 0.5–1%), the sensitivity might be relatively low (70–80%), translating into a rate of false negatives of at least one out of five cases [44, 45]. This phenomenon is believed to be linked to the amounts of ctDNA shed into the plasma, which could be lower when tumour burden or shedding capacity is limited [6, 46].

The main limit of the narrow approach is related to its possibility to interrogate a very limited number of loci and are intrinsically unable to provide comprehensive molecular characterisation.

In parallel, data on PCR-based techniques for plasma detection of other important druggable gene alterations, such as *ALK* rearrangements, are very limited, they show very low sensitivity rates for fusion genes and prospective validation studies are not available yet [47–49].

On the other hand, high-throughput NGS-based multi-gene tests can be performed in order to interrogate in a single workflow several genomic alterations, including single nucleotide variant (SNV), small insertions and deletions (indels), gene rearrangements, CNVs, and define their variant allelic frequency (VAF). The amount of information a single NGS analysis can provide is strictly linked to the size of the gene panel analysed. Moreover, the sequencing coverage could significantly affect costs, turn-around times and also the sensitivity of NGS assays [50]. Historically NGS-based methods have been considered less sensitive than PCR-based ones; however, recent studies using ultra-deep NGS have described a similar sensitivity for the detection rate of certain driver mutations [51]. High analytical specificity is the main feature also of NGS-based genotyping assays, with a positive-predictive value of over 99%. In this context, the phenomenon of clonal hematopoiesis is noteworthy: some mutations detectable in the ctDNA are attributable to the DNA of white blood cells, rather than to tumour genetic material [52]. This issue might affect the interpretation of the test, especially when the VAF of the genetic variant called by the test is low; therefore, some assays have now incorporated paired sequencing of ctDNA and white blood cell DNA to overcome this hurdle [51].

Overall, NGS-based multi-target approaches have the advantage of allowing the simultaneous analysis of multiple genetic alterations, but they are more expensive and technically demanding and they probably require centralisation of testing for clinical use.

## Liquid biopsy as the challenge of today

**Fig. 2:**
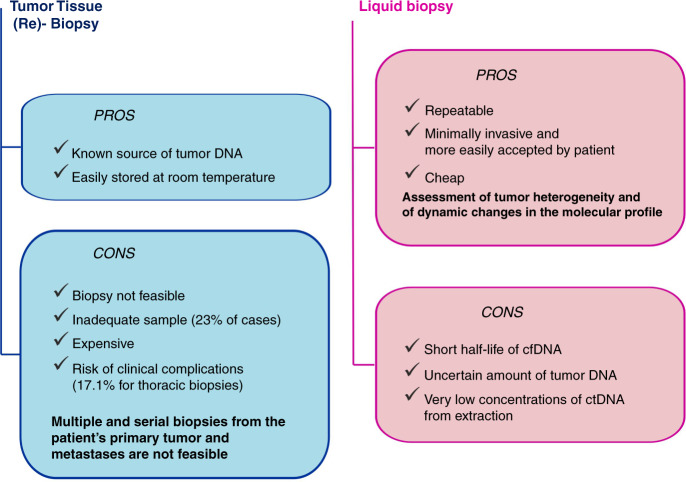
Pros and cons of tissue and liquid biopsy. A simplified summary of potential advantages and limits of liquid biopsy, when compared to standard approach (tissue biopsy).

To date, undoubtedly, tissue biopsy represents the gold standard for lung cancer diagnosis and cannot be replaced by liquid biopsy. However, scarcity of tumour tissue is common, with over 40% of patients needing more than one procedure in order to achieve a definitive diagnosis of lung cancer [53]. In tissue, the failure rate of adequate molecular characterisation, essential for the proper treatment of lung cancer, revolves around 30% of cases [5] (Fig. 2). Over time, the number of molecular information needed is increasing. ESMO guidelines currently recommend testing at least *EGFR* mutations, *BRAF* mutations, *ALK* fusions, *ROS-1* fusions, *MET* exon 14 skipping mutations, *RET* rearrangements and PD-L1 expression levels in non-squamous advanced NSCLC [54]. This panel could be further implemented considering *KRAS* mutations, *HER2* mutations, *MET* amplification and *NTRK* rearrangements [54].

The first application of liquid biopsy in clinical practice concerns *EGFR*-mutated advanced NSCLC (Fig. 1). Testing *EGFR* mutation in plasma at baseline when tissue analysis is not feasible has been accepted in clinical practice to select patients for first-line treatment with EGFR-TKIs [54].

**Fig. 3:**
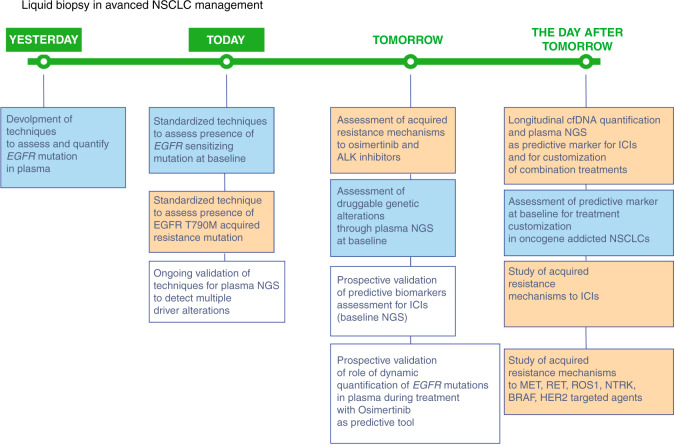
A summary of past, present and future applications of liquid biopsy in advanced NSCLC management. Blue colour is used for static applications of liquid biopsy in clinical practice, orange colour is used for dynamic applications while in white squares validation tests are included.

The second clinical application of liquid biopsy in *EGFR* mutation-positive NSCLC is the evaluation of the most common acquired resistance mechanism to first and second-generation EGFR-TKIs (erlotinib, gefitinib and afatinib): T790M mutation [55, 56] (Fig. 3).

In AURA3 trial, demonstrating the effectiveness of osimertinib in patients with acquired *EGFR* T790M mutation, tissue re-biopsy was mandatory to assess T790M mutational status, but a pre-defined subgroup analysis in patients with T790M positive in plasma confirmed the clear superiority of osimertinib over chemotherapy in this subgroup of patients [15]. A pivotal retrospective analysis in patients treated with osimertinib in phase I AURA study demonstrated that the sensitivity of detecting *EGFR* mutation in plasma by using BEAMing technology, a PCR-based method, was 82% for exon 19 deletion and 86% for L858R mutation and confirmed the predictive role of T790M mutation detected in plasma [57]. The sensitivity for *EGFR* T790M mutation detection in plasma can vary according to the technology, but might reach 93% with ddPCR [58, 59].

In addition to technical issues, *EGFR* T790M detection rate might be associated with different biological reasons, being tumour heterogeneity and low tumour burden. Liquid biopsy in this context could also provide additional information: if T790M ratio to activating mutation in tumour may correlate with the response to osimertinib [57, 60], the allele frequency of *EGFR*-activating mutations and the ratio T790M/sensitising mutation in plasma have been associated with response to osimertinib [61].

Recently, a relatively large real-world retrospective analysis has confirmed the correlation between T790M status and tumour burden. Probably, for this reason, patients with T790M positive in plasma had a worse disease control rate (DCR) when compared to negative ones [62].

The presence of a high-prevalence resistance mechanism to first- and second-generation EGFR-TKI led to the optimisation of liquid biopsy in order to detect *EGFR* T790M in cfDNA. International guidelines include liquid biopsy as the initial test for detection of T790M mutation in patients with evidence of TKI resistance. However, owing to the limited sensitivity of liquid biopsy [63], when a negative result is obtained, tissue biopsy is necessary, whenever feasible [54].

NGS assays are also approved as companion diagnostics to identify *EGFR* mutations and *ALK* rearrangements that predict benefits from EGFR-TKIs and ALK-TKIs, respectively. However, the most appealing application of NGS in liquid biopsy is the study of additional druggable alterations (Fig. 3). The first study on 93 consecutive patients with advanced NSCLC with insufficient or inadequate tumour samples for standard molecular characterisation showed how NGS ctDNA genotyping with Guardant360 CDx was able to detect potentially actionable genomic alterations in 53 cases [64]. Twelve patients received matched therapies, deriving significant clinical benefits [64]. Another large experience reported the use of Guardant360 CDx to genotype over 8000 advanced NSCLC patients on ctDNA and 879 were considered as “undergenotyped” after tissue-based analyses, i.e. not evaluable for all guideline-recommended genetic alterations [65]. We also recently described a real-life experience with the prospective evaluation of liquid biopsy genetic screening using Guardant360 CDx in over 200 advanced NSCLC patients, already tested in tissue with standard molecular techniques, and 42 cases were tested positive for previously undetected druggable alterations. The administration of matched targeted agents was associated with improved outcomes [22].

All these experiences underline how the application of targeted-gene NGS panels to analyse ctDNA could actually improve clinical practice, allowing the detection of actionable genomic alterations and the consequent administration of a tailored systemic anti-cancer treatment. However, one of the most relevant issues to address is the assessment of gene fusions in liquid biopsy. Their detection by the NGS-based cfDNA analysis is challenging and the sensitivity of detecting in cfDNA is currently debated [66, 67]. Moreover, recent reports describe an incidence of detection of gene fusions in plasma samples lower than expected in NSCLC [68, 69]. To this date, the largest, prospective, real-world study evaluating the clinical relevance of liquid biopsy in *ALK* and *ROS-1* rearranged advanced NSCLC patients, showed a sensitivity rate of 67% for the detection of such fusions by amplicon-based NGS [70]. Gene fusions arise from the inter-chromosomal or intra-chromosomal conjunction of different introns. For this reason, they are difficult to detect by DNA-based NGS, especially when the introns are large, or contain repetitive sequences, and genes present several fusion partners [71, 72]. In this context, analysis of the circulating cell-free (tumour) RNA (cfRNA/ctRNA) is not influenced by the previously described limitations and could complement ctDNA for the investigation of fusion gene abnormalities. In fact, analysis of cfRNA in circulation or embedded in vesicles or tumour-educated platelets (TEPs) has shown widely applicability in the detection of several cancer-associated aberrations [73, 74]. The sensitivity of cfRNA-based assays could reach over 77% [67]; however, its clinical implementation is to be improved and needs standardisation of liquid biopsy workflow and optimisation of pre-analytical conditions.

Overall, the availability of liquid biopsy and NGS techniques for ctDNA analysis are already affecting our clinical approach to advanced NSCLC management. In particular, the great amount of information obtained by using NGS in clinical practice and the integration of off-label treatments in therapeutic pathways are one of the main challenges for modern oncology and should be managed by a specifically organised multidisciplinary team, termed molecular tumour board [75]. In the next future, the optimisation of standardised techniques could also let us face other questions about the possibility of treating patients according to liquid biopsy analysis in the absence of tissue-based genetic characterisation and even in the absence of a sure pathological diagnosis. Anyway, genetic information obtained from liquid biopsy analysis does not provide a complete molecular characterisation. In this context, the tissue biopsy is the optimal and not replaceable source for analysing the expression of PD-L1 for routine use in clinical practice and permit to investigate the role of tumour immune microenvironment. Consequently, in our opinion, the integration of tissue and plasma characterisation represents the preferred approach for advanced NSCLC management in the next future.

## Liquid biopsy in the clinical practice of tomorrow

Based on available evidence, liquid biopsy applications that we feel almost ready for prime-time are the detection of acquired resistance mechanisms to EGFR- and ALK-TKI (Fig. 3). On the other hand, growing evidence is available about the potentiality of blood-based TMB (bTMB), even though its clinical applications have not been clearly defined yet.

While the detection of T790M resistance mutation in plasma has led to the spread of liquid biopsy in clinical practice, the phase III FLAURA trial assessed the superiority of osimertinib over gefitinib or erlotinib in first-line setting [76, 77] and established osimertinib as standard treatment for first-line therapy in *EGFR*-mutated advanced NSCLC, thus opening new perspectives for liquid biopsy applications.

Acquired resistance mechanisms to osimertinib are more heterogeneous, than those to first- and second-generation EGFR-TKIs.

Most information stemmed from studies in patients treated with osimertinib in second- or third-line settings. The revision of these studies shows the increased role of liquid biopsy in this setting. Acquired resistance mechanisms to osimertinib can be classified as on- or off-target. Multiple *EGFR* acquired mutations have been identified, but the most frequent is *EGFR* C797S mutation, occurring at osimertinib binding site [78–80]. Off-target alterations are more frequent than in acquired resistance to first- and second-generation TKIs. The most frequent one is *MET* amplification, concerning about 20% of cases [78, 81, 82]. In patients progressing to osimertinib, the persistence of T790M mutation has also been associated with improved outcomes [83, 84]. Among patients enrolled in AURA3 trial, 83 had paired plasma samples for comparing NGS analyses by using Guardant360 at baseline and at the time of progression to osimertinib: 15% of patients acquired an *EGFR* mutation, mainly C797S, while *MET* amplification was detected in 19% of patients [85]. Smaller studies were based on tissue samples and they confirmed the persistence of *EGFR* founder mutation at the time of progression to osimertinib and main resistance mechanisms found through liquid biopsy, but they underlined the role of histological transformation [83, 86]. In two studies analyzing tissue re-biopsies, three out of 32 and six out 41 patients developed histological transformations [83, 86].

Acquired resistance mechanisms to first-line osimertinib are less investigated and they might be different from those to second-line osimertinib. To this extent, among patients enrolled in FLAURA trial, 91 were evaluated by liquid biopsy (NGS Guardant360 73 gene panel or Omni 500 gene panel) and the most common described mechanism was *MET* amplification (15%), followed by *EGFR* C797S mutation (7%); acquired *HER2* amplification, *PIK3CA* and *RAS* mutations were also described [87]. The first tissue-based series describing first-line osimertinib resistance mechanisms included 27 patients and highlighted 15% of histological transformation [88]. The study of acquired resistance mechanism to first-line osimertinib is one of the key issues in the way to further improve the outcome of *EGFR*-mutated patients.

No targeted treatments are currently approved for osimertinib acquired resistance, although several clinical trials are ongoing, especially in the setting of *MET*-amplified patients. Different strategies are being tested in the context: amivantamab and lazertinib combination (Chrysalis-2, NCT04077463) [89], osimertinib plus savolitinib association (TATTON, NCT02143466) [90, 91] and tepotinib plus osimertinib combination (INSIGHT 2, NCT03940703). These ongoing trials require mandatory tissue re-biopsy, but include also systematic plasma collection, which could validate liquid biopsy for *MET*-positive acquired resistance detection. Interestingly, INSIGHT 2 also include a cohort of patients treated with positive liquid biopsy and negative/not evaluable tissue biopsy [92]. In this context, small experiences evaluating liquid biopsy-based strategy to assess *MET* amplification showed a high level of concordance with tissue (91.67%), with a sensitivity rate of over 85% [93].

Other acquired resistance mechanisms detectable through liquid biopsy have potential clinical application in the next future and wide NGS analysis in plasma at the time of progression is able to identify additional druggable alterations, such as *ALK* and *RET* rearrangements and *BRAF* V600E mutations, and strategies with a combination of targeted agents have been reported [94, 95].

Another potentially druggable acquired resistance mechanism concerns resistance to second-line osimertinib through the development of *EGFR* C797S is in *trans* with the T790M mutation cells. A combination of first- and third-generation EGFR-TKIs might be effective, while fourth generation EGFR inhibitors are under development. Since *cis* mutations are found to be resistant to EGFR inhibition [96], the potential of plasma NGS to detect if C797S tertiary mutations are in *cis* or *trans* might of potential clinical usefulness [96, 97].

Small-cell lung cancer (SCLC) transformation has been described as another resistance mechanism, accounting for 3 to 10% of all the EGFR-TKI-resistant cases [98]. However, this might be underestimated, due to the absence of re-biopsy at the time of progression. The most common mutations associated with SCLC transformation included *TP53*, *RB1* and *PIK3CA*. Small experiences, applying complementary testing of tumour tissue and liquid biopsy, showed how ctDNA analysis could suggest neuroendocrine evolution, when typical SCLC-associated genetic alterations become detectable [99, 100]. Squamous cell carcinoma transformation has also been described in different case reports after EGFR-TKI treatment [101]. Such phenomenon seemed to be linked to the emergence of *PTEN* alterations and with considerable genomic complexity, including co-occurring gene mutations, amplification and chromosomal rearrangements [88]. In this context, tissue re-biopsy appears to be irreplaceable for detecting histologic transformation [102].

Overall, acquired resistance mechanisms to osimertinib are heterogeneous and require screening with methods able to detect multiple genetic alterations. NGS appear to be the best approach in this setting, but requires a relatively high amount of DNA, not always easy to obtain by tissue re-biopsy. Nevertheless, the relatively high fraction of histological transformation underlines the importance of paired tissue re-biopsy to get as much information as possible in prospective trials on acquired resistance to osimertinib.

One of the challenges for the future of our patients is to validate in clinical practice NGS panels including the most frequent genetic alterations related to acquired resistance to osimertinib, considering their potential therapeutic impact. This panel could then be routinely used and associated with tumour re-biopsy when feasible.

Prospective systematic analyses of resistance mechanisms to osimertinib are ongoing: MELROSE (NCT03865511) and ELIOS study (NCT03239340) are assessing genetic tumour profile in tissue and plasma at the time of disease progression to first-line osimertinib. On the other side, ORCHARD trial (NCT03944772) is an open-label, biomarker-directed phase II platform study, including targeted treatment options for patients progressing on first-line osimertinib according to molecular characterisation performed in tissue re-biopsy.

One of the most important settings displaying the huge potentiality of liquid biopsy is *ALK*-rearranged disease. Several ALK-TKIs have been approved for advanced NSCLCs harboring *ALK* translocations. Crizotinib, a first-generation ALK-TKI, was the first approved ALK inhibitor, while second-generation TKIs, ceritinib, alectinib, brigatinib and ensartinib, were initially evaluated for patients progressing to crizotinib and subsequently moved to first-line following phase III trials [103]. Lorlatinib, a third-generation ALK-TKI, demonstrated to be active in patients progressing to first- and second-generation ALK-TKI and later to be superior over crizotinib in first-line [104, 105].

While the *ALK*-rearranged treatment scenario is enriching, several points still need to be improved to optimise *ALK*-rearranged treatment options: no selection criteria are currently available to choose among the different ALK-TKIs available in the first-line setting. Different variants of *ALK* rearrangements, potentially affecting the response to ALK-inhibitors, are not routinely tested in clinical practice and, above all, molecular analysis of acquired resistance mechanisms are not routinely evaluated and are not currently leading treatment choice at the time of progression.

Liquid biopsy has the potential to provide tools to fill these gaps and its role might be even more relevant than in *EGFR*-mutated disease, due to the heterogeneity of resistance mechanisms.

NGS technique permits the detection of *ALK* status at baseline and might be useful when tissue biopsy is not adequate for immunohistochemistry (IHC) and fluorescent in situ hybridisation (FISH) analysis, but also to detect different kinds of *ALK* rearrangements, including those not detectable by using FISH method. This approach has been investigated by the BFAST trial, a phase I/II study screening 2,219 patients with FoundationOne Liquid CDx assay and 87 out of 119 *ALK*-positive patients received alectinib and the radiological response rate was 87.4% [106]. This was the formal confirmation of the predictive role of *ALK* rearrangements detected in plasma through NGS, while a concordance of 91% between tissue and plasma evaluation was shown in patients enrolled in eXalt2 phase I/II trial investigating safety and efficacy of ensartinib [107].

Anyway, the most important applications of liquid biopsy in *ALK*-positive advanced NSCLC are likely to concern the study of acquired resistance to ALK inhibition.

Acquired resistance mechanisms to ALK-TKIs are both on-target and off-target, but on-target mechanisms are highly heterogeneous, thus including different *ALK* mutations and *ALK* amplifications. First analyses were retrospective and performed in tissue small series at progression to crizotinib using targeted approaches; they showed on-target alterations in less than one-third of cases and described different *ALK* acquired mutations. Off-targets alterations included *EGFR* mutation/amplification, *KIT* overexpression, *KRAS* mutation and *MET* amplification [108–110]. Later, a pivotal study performed wide NGS on re-biopsies from 51 patients progressing on crizotinib and second-generation TKIs. Interestingly, the study enhanced different patterns of acquired resistance mechanisms following first- or second-generation TKI. Notably, a higher fraction of patients progressing to second-generation TKIs developed G1202R resistance mutation, associated with sensitivity to lorlatinib [111].

More recent studies on acquired resistance mechanisms to ALK-TKIs were performed mainly by using liquid biopsy and wider use of NGS analysis increased the level of information obtained. In particular, these studies showed heterogeneity of *ALK* mutations detected, increased number of mutations acquired after second- and third-generation TKIs, potential underestimation of resistance mutations in tissue and potential predictive role of *ALK* mutations in patients treated with lorlatinib after at least one second-generation TKIs [112–114]. Among off-target mechanisms, *MET* amplification has been demonstrated to be more likely when patients are treated with new-generation TKIs in first-line setting, potentially opening new treatment perspectives for the next future [110].

Finally, the aim of evaluating resistance mechanisms to ALK-TKIs is to apply a tailored approach to further-line therapies. In this context, a systematic approach is under evaluation in the NCI-NRG ALK study (NCT03737994), a phase II study including tissue and plasma genotyping by NGS after progression on a second-generation TKI (ceritinib, alectinib, ensartinib and brigatinib). Treatment options at the time of progression are designed according to molecular characterisation: type of acquired single or compound resistance mutations, *MET* amplification, absence of resistance mutations.

In parallel with the discovery of activity and efficacy of new targeted agents against other driver genetic alterations [115–123], translational research focuses on the study of acquired resistance mechanisms. In this context, liquid biopsy seems the preferred approach associated with tissue re-biopsy, whenever feasible. In particular, the first reports are available concerning acquired resistance alterations developed after treatment with *KRAS* [124], *MET* [125] and *RET* inhibitors [126, 127]. Both on-target and off-target alterations were described, thus opening further therapeutic perspectives to be developed in the next future.

Another application of liquid biopsy investigated in advanced NSCLC and potentially not so far from clinical practice is bTMB evaluation. Several phase III trials demonstrated the role of ICIs, alone or in combination strategies, in the management of the vast majority of advanced NSCLC [54, 128], but response and duration of clinical benefit following ICIs are highly heterogeneous and even potential detrimental effects have been depicted [129]. Treatment selection is needed in order to improve the treatment approach and minimise the cost of cancer therapy and useless toxicity. TMB is defined as the total number of non-synonymous somatic mutations per Mb in the coding region of the cancer genome and it is associated with the neoplastic production of neoantigens to be recognised by T immune cells [130]. For this reason, TMB has the potential to mirror the capacity of tumour cells to elicit an immune response and it has been correlated to response to ICIs [131]. TMB is optimally determined by using NGS to sequence all the protein-coding regions of the genes in the genome, a technique called whole-exome sequencing (WES). However, WES is highly expensive, requires a high amount of good quality DNA and high specific expertise for results analysis. Estimates of TMB can be given by sequencing a more limited number of genes and selected gene panels have been validated for this purpose [131]. The predictive role of tissue TMB (tTMB) was assessed in several retrospective studies, by using it both as a continuous variable and as a categorical one, by defining different cut-off points [132–135]. Efficacy by tTMB was also prospectively evaluated among patients treated with nivolumab plus ipilimumab in Checkmate 568 phase II trial as a secondary endpoint [136]. Promising results of this trial were not confirmed in larger trials. In the Checkmate 227 phase III trial, tTMB was tested by using the same method and cut-off value but its predictive value was not confirmed when considering overall survival as outcome endpoint; [137, 138] on the other hand, no predictive role has been observed among patients treated with chemo-immunotherapy [139].

Smaller evidence is provided concerning the potential clinical value of bTMB. Pivotal retrospective analysis indicated the predictive role of bTMB by using three different cut-off values and evaluating two independent groups of patients treated with second-line atezolizumab versus docetaxel [31]. Prospective data on the potential predictive role of bTMB are available in patients treated with ICIs in first-line setting atezolizumab or combination treatments including durvalumab plus tremelimumab or chemotherapy [29, 140–143].

Overall, even though data about the potential predictive role of tTMB are still considered controversial and far from clinical applicability; technical limits to perform tTMB in small biopsies suggest that bTMB could be more promising and deserves further validation in clinical practice. Standardisation of technologies, cut-off definition and significance of cases with no genetic alterations detected in plasma need to be pursued, in order not to lose the opportunity to exploit biological knowledge and the potential role of TMB in this setting [131, 144].

The potential relevance of TMB is also mirrored by the performance of harmonisation studies comparing several assays in order to face over-mentioned technical issues [131, 145–147].

In addition to bTMB, NGS analysis in plasma permits individuate genetic alterations associated with resistance to ICIs, such as *LKB1*/*KRAS*, *LKB1*/*KEAP1* or *LKB1*/*KEAP1*/*SMARCA4* co-mutations [23, 148, 149].

## The day after tomorrow: what we are looking for

Despite the increasing amount of evidence available, what we currently know is likely only the tip of the iceberg of future applications of liquid biopsy in advanced NSCLC.

What we are looking for is to have as much information as possible at diagnosis, in order to optimise treatment options and get predictive information, but also to monitor biological changes of the disease during treatment.

To this extent, technical improvements are under evaluation, to permit adequate static and dynamic analysis of multiple genetic alterations such as CNVs and genomic rearrangements [150].

In the setting of oncogene-addicted disease, the presence of co-mutations found in plasma at baseline and the persistence of *EGFR* mutations in plasma after a short course of treatment could address the choice of combination treatment, being potentially associated with shorter benefit from targeted agents [62, 151, 152]. For example, the presence of *TP53* mutations, or *PIK3CA* mutations, *RB1* alterations, FAT tumour suppressor homolog 1 (*FAT1*), or ATP-binding cassette sub-family B member 1 (*ABCB1*) mutations in *EGFR*-mutated patients, potential negative predictive and prognostic biomarkers, could lead to choose to administer EGFR-TKIs combined with chemotherapy (and/or antiangiogenic agents) [151–153]. In this context, future applications of liquid biopsy are likely to concern the study of primary resistance to treatment in order to optimise treatment personalisation, also taking into account upcoming treatment options.

On the other side, the systematic evaluation of genetic alterations in plasma at baseline and at the time of progression has the potential to provide information concerning predictive markers and acquired resistance mechanisms in a shorter period compared to what has happened before by using retrospective evaluation in tissue. This approach should be encouraged early in the development of new targeted agents and pursued through prospective studies in patients treated according to clinical practice.

In the field of immunotherapy also the liquid microenvironment deserves further study as a potential predictive marker. As a matter of fact, pre-treatment T-cell receptor repertoire metrics could predict response to pembrolizumab and immune-related toxicity [154].

By the way, the role of liquid biopsy could not be limited to baseline evaluation. Recent experiences demonstrated a potential role for quantitative changes in tumour-associated mutations during treatment as able to mirror changes induced by ICIs and therefore anticipate outcomes to ICIs [26, 155]. Our first experience has been obtained by tracking *KRAS* mutations by ddPCR, but confirmation by using NGS is ongoing and, interestingly, early evaluation in plasma could be able to anticipate potential detrimental effects of ICIs [26].

On the other hand, the interest in liquid biopsy in lung cancer surely goes towards early-stage disease. For surgically resected patients, a reliable risk assessment evaluation is one of the major needs, also taking into account the new upcoming treatment options in the adjuvant settings. Minimal residual disease (MRD) refers to the persistence of residual cancer cells after treatment, in the absence of evidence detectable through conventional investigation, such as medical imaging [156]. The detection of tumour genetic material in plasma is a promising tool to assess MRD and information stemming from different sources of genetic material could optimise the model. Several trials are currently evaluating MRD assessment in NSCLC patients treated with curative intent, including the phase III MERMAID-1 (NCT04385368) [157] and MERMAID-2 trial (NCT04642469) [158].

Besides, liquid biopsy has potential applications even in the field of lung cancer screening. If low-dose computerised tomography (LDCT) has demonstrated a role in lung cancer screening for heavy-smoker patients [159, 160], the detection of false-positive cases is one of the major concerns [161]. To overcome this aspect, a recent study proposed a cfDNA-based machine-learning method, termed lung cancer likelihood in plasma (Lung-CLiP) to discriminate early-stage lung cancer patients from risk-matched controls [162]. Liquid biopsy approaches tested for application in the field of lung cancer screening include the analysis of cfDNA methylation to distinguish between non-tumour and tumour cfDNA [163], the study of plasma microRNA [164] or the analysis of plasma metabolites through mass spectrometry [165].

Future directions for liquid biopsy in lung cancer are also related to the study of other analytes in plasma beyond ctDNA, which could open new perspectives in the next future. CTCs are not routinely used in clinical practice and in main translational studies planned for large clinical trials, due to the low number of CTCs usually detected in NSCLC. In addition, the detection and analysis of CTC, although biologically extremely fascinating, has major technical challenges and require high specific expertise and more expensive approaches, when compared to ctDNA, and standardised pre-analytical procedures are not available yet [166, 167]. CTCs might have negative prognostic value in early-stage disease; moreover, they could be potentially useful for the study of PD-L1 expression. Their heterogeneous phenotype and genotype yield CTCs analysis advantageous to investigate genetic heterogeneity through a dynamic approach. Finally, the development of CTC-cultures and CTC-derived xenograft could be of help in the research field and even in personalisation of the treatment approaches [166, 167]. As mentioned above, other analytes with potentially interesting applications are TEPs, being a potential source of abundant high-quality RNA. Extracellular vescicles are also a source of more abundant genetic information when compared with ctDNA and they are characterised by high stability, even though DNA extraction procedures are technically more complex. Finally, microRNA are small non-coding RNA, frequently deregulated in tumours and they can be detected and analysed in plasma, even though several technical issues have to be faced before validation for clinical practice [168–170].

In conclusion, liquid biopsy has huge potentialities in advanced NSCLC treatment and systematic prospective evaluation of its role, in parallel to validation of techniques specifically designed for clinical needs, are one of the main challenges to improve advanced NSCLC management in the next future.

## Data Availability

Not applicable.
